# The Origin and Understanding of the Incretin Concept

**DOI:** 10.3389/fendo.2018.00387

**Published:** 2018-07-16

**Authors:** Jens F. Rehfeld

**Affiliations:** Department of Clinical Biochemistry, Rigshospitalet and University of Copenhagen, Copenhagen, Denmark

**Keywords:** gastrointestinal hormones, incretin, GIP, GLP-1, history of incretin

## Abstract

Gastrointestinal hormones that stimulate insulin secretion at physiological concentrations are incretins. This concept has recently attracted considerable attention in the wake of drugs developed from the gut hormone GLP-1 (glucagon-like peptide-1) for diabetes therapy. But the renewed enthusiasm has also restricted the concept to just two hormones, GLP-1 and GIP (glucose-dependent insulinotropic polypeptide). The purpose of the present overview is two-fold: First to tell that the incretin concept is far from new. It has a more than a century long history full of ups and downs. Second, that the incretin concept may now have become too narrow. Thus, it is likely that incretin comprises additional gastrointestinal hormones, which interact with GIP and GLP-1 during normal meals containing protein, fat and complex carbohydrates (and not just pure glucose). Such broader incretin concept may stimulate development of novel gut hormone-derived drugs.

## Introduction

In gastrointestinal endocrinology, the concept of incretin is today highly topical and generally applied to two distinct gut hormones with technical acronymous names: GIP (originally “gastric inhibitory polypeptide,” later renamed “glucose-dependent insulinotropic polypeptide”) and GLP-1 [“glucagon-like peptide 1,” now in its truncated (7–36) form]. Of course it is more idiomatic to use the single word “incretin” instead of two acronyms. Moreover, many younger scientists and physicians today consider incretin a rather novel and fashionable concept surfacing in the wake of the development of GLP-1-derived drugs for treatment of type 2 diabetes mellitus, a view furthered by the growing business for the diabetes-related pharma industry. In other words, there is at present a marked focus on the two above-mentioned hormones in gastrointestinal endocrinology. To this end, there are also articles which are ostensibly dealing with gut hormones but mainly report about GLP-1 and/or GIP, sometimes accompanied by measurements of a third gut hormone, PYY (“peptide tyrosyl-tyrosyl”), or measurements of ghrelin [see for instance (1–4)]. These articles contribute to the picture that gastrointestinal endocrinology today is essentially about GIP and GLP-1.

It is exciting that gut hormones are now used as targets for development of drugs for major diseases with large numbers of patients. But this is in fact what has been attempted for more than a century. Likewise, the incretin concept as such is in some respects more than 140 years old. But with the present conceptualization to just two hormones, incretin may lose aspects of its meaning and understanding of what gastrointestinal endocrinology is fundamentally about. Equally unfortunate, initiatives to develop additional relevant drugs may also be lost with today's narrow view on incretin.

In this situation, a review on the origin and early phases of gastrointestinal endocrinology leading to the incretin concept may be pertinent. The report here may hopefully also pave the way for a fuller and more relevant understanding of the biology of gut hormones, and at the same time give due credit to pioneers in the incretin story.

## Definition of incretin

Incretin is a word and concept constructed for a gut hormonal factor assumed to supplement secretin in the effect on pancreatic secretion. Thus, while secretin stimulates the secretion of water and bicarbonate from the *exocrine* pancreatic cells (5, 6), it has been suggested from the beginning that (an) other gut hormone(s) would stimulate the internal or *endocrine* secretion from pancreatic islet-cells (6, 7). Literally, it was the Belgian physiologist Jean La Barre who coined the word “incrétine” in 1932 (8). Consequently, the original definition suggests that any gut hormone which under physiological circumstances stimulates or contributes to the stimulation of the secretion of pancreatic hormones [insulin, glucagon, PP (pancreatic polypeptide), and pancreatic somatostatin] is an incretin.

The physiological context is of course important. Since the function of hormones in the digestive tract fundamentally is to facilitate digestion and subsequent absorption and metabolism of food elements, the incretin activity is linked to the gastrointestinal processing of ordinary meals. Hence, the original incretin definition challenges unphysiological loadings, such as intake of large amounts of for instance pure glucose or other pure chemicals.

## The history of incretin

1850–1900: The mental framework for the idea of incretin dates back to the second half of the nineteenth century, where European physiologists began to focus on the mechanisms of the external and internal secretion of the pancreas (5, 9–13). It was in this period that Mering and Minkowski showed that the pancreas was the site of origin for diabetes mellitus (14), and where Claude Bernard tried to explain the fact that significantly larger amounts of glucose can be given orally than intravenously without glucosuria. Hence, Claude Bernard suggested that the liver takes up most of the oral glucose during the first portal circulation in order to prevent hyperglycemia (15). This explanation had supporters up to the 1950's (16).

1900–1960: A decisive breakthrough came in 1902, with Bayliss' and Starling's hallmark discovery of secretin (5, 6) that founded not only gastrointestinal endocrinology, but also endocrinology in general. The discovery of secretin was also the background for Starling's Croonian lecture from 1905 (6), in which he coined the word *hormone* (from Greek “hormoa”: I arouse to activity). According to Moore et al. (7), the discovery of the first hormone, secretin, also led Starling to suggest the possibility that the duodenal mucosa in addition to secretin produces another hormone that stimulates the internal secretion of the pancreas. Moore et al. immediately tested Starling's hypothesis by oral administration of extracts of duodenal mucosa to three recently diagnosed diabetes patients (7). In hindsight, the results of such oral intake were of course negative and inconclusive, because protein- and peptide-hormones are proteolytically degraded in the stomach. But the idea of incretin was born more than 110 years ago.

After the Banting & Best discovery of insulin in 1922 (17), new attempts were taken to examine extracts of the duodenal mucosa and their influence on blood glucose concentrations (18–21). These results were also conflicting and inconclusive (21), which in retrospect cannot surprise. But promising results were nevertheless obtained by La Barre and Still, who in 1930 reported that they in the *in vitro* processing of duodenal extracts had obtained two interesting fractions: One with crude secretin, which in sophisticated cross-circulation experiments in dogs stimulated the secretion from the exocrine pancreas, and another which lowered blood glucose concentrations without effect on the secretion of the exocrine pancreas (22). They also suggested that the glucose-lowering effect was due to stimulated insulin secretion (22). Then in 1932—as mentioned above (8)—La Barre presented the name, incretin, together with suggestions for treatment of diabetes mellitus with incretin (23). Strictly speaking, the articulated idea of incretin-therapy for diabetes is thus nearly a century old.

After La Barre's hallmark contributions to the incretin story (8, 21–23), the Austrian Hans Heller also prepared an extract of the duodenal mucosa, which he in 1935 reported to lower blood glucose concentrations—even after oral administration to rabbits and man (24). Heller named the active factor in his extract “duodenin.” His results, however, have not been followed up, and the unspecific name duodenin was rapidly forgotten. Then, synchronously with the onset of the Second World War, the incretin-idea in general suffered an almost deadly blow from the Chicago-school of gastrointestinal endocrinology (25–27). The school was founded by Andrew Ivy, well known from the discovery of cholecystokinin (CCK) in 1928 (28). After three publications in rapid succession 1939–40 about acidification of the duodenum in dogs at various blood glucose concentrations (25–27), Ivy et al. concluded that the existence of an incretin is unlikely. This opinion was neither challenged nor contradicted during World War II and in the two first post-war decades. On the contrary, the younger Ivy-pupil and -successor as spokesman for American gastrointestinal endocrinology, Morton Grossman, emphasized in a comprehensive high-impact review in 1950 the scepticism against the incretin concept (29). But as time has shown, Ivy and co-workers were wrong. They drew false-negative conclusions of their experiments that in fact only showed that secretin in dogs is without significant effect on insulin secretion. Nevertheless, their publications paralyzed further ideas and initiatives about incretin for a quarter of a century.

1960–2000: The year 1960 witnessed a major breakthrough for biomedicine and not least endocrinology. It virtually changed the world and revitalized the interest in incretin. It was the invention of the radioimmunoassay (RIA) by Berson and Yalow (30). The RIA technique allowed for the first time in a fairly uncomplicated, but accurate manner measurement of molecules present in pico- to even femtomolar concentrations. A world of biologically active substances, including peptide hormones, circulates in plasma in those concentrations. Therefore, RIA methods expanded the dimensions of much biological and medical research. Not least in basic and clinical endocrinology, because hormones are defined by their circulation in blood. For good reasons, the RIA technology was first applied to insulin (30). Therefore, the method was immediately embraced by endocrinologists and diabetologists studying pancreatic endocrine secretion and diabetes mellitus. Only one year later, RIA measurement of glucagon was launched by Unger et al. (31). And each year during the following decades bursted with novel RIAs for known and new pancreatic and gastrointestinal hormones (32).

The possibility of direct and reliable measurements of insulin in plasma soon reopened the incretin question. In 1964, laboratories in London, UK [McIntyre et al. (33)] and Denver, US [Elrick et al. (34)] independently showed that oral glucose provokes a considerably larger insulin response than intravenous glucose, even at similar blood glucose concentrations. Hence, the gut harbors indeed insulinotropic hormonal factors. Or in other words, the incretin mechanism exists. The reports of McIntyre and Elrick et al. catalyzed new incretin studies in man, which followed three lines.

**Figure 1:**
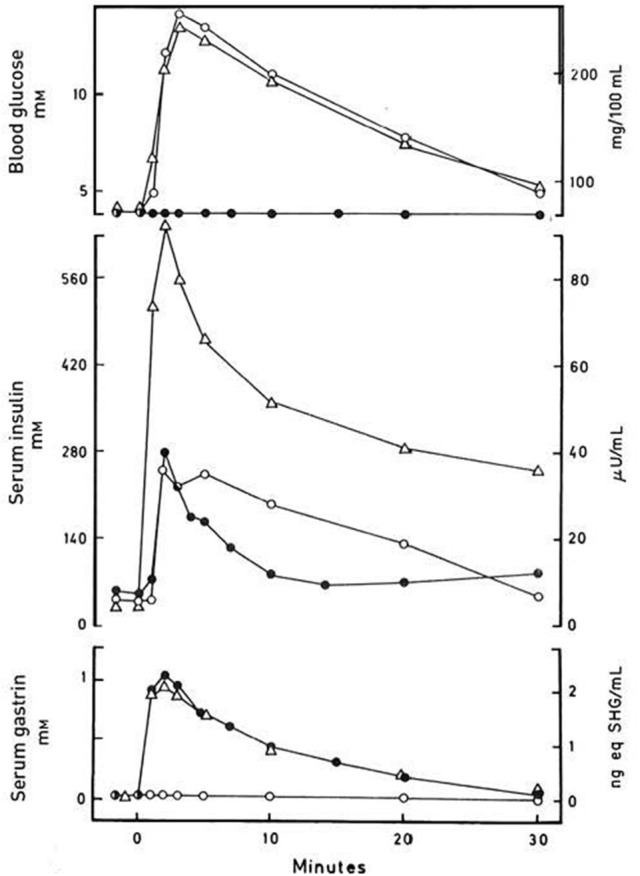
Blood glucose, serum insulin and serum gastrin concentrations in a normal subject after i.v. injection of synthetic human gastrin-17 (SHG), 250 ng kg^−1^ body weight (•), after i.v. injection of 25 g glucose (o), and after a synchronous i.v. injection of both 250 ng kg^−1^ gastrin-17 and 25 g glucose (Δ). Data from Rehfeld & Stadil (44).

One line focussed on *in vivo* development of methods for quantitation of the glucose-induced incretin effect. Here, Perley and Kipnis (35) showed that the incretin part of the insulin response to oral glucose in man constituted more than half, later confirmed to be probably two thirds or more of the insulin response in healthy people, though smaller, with high age and some gastrointestinal diseases (36, 37). Another line examined the incretin effect of the then known troika of gastrointestinal hormones (secretin, gastrin, and CCK) that could be obtained in more or less pure forms in the mid-1960's (38–43). These studies were later reinvestigated with pure, synthetic peptides (44, 45). The immediate results of the studies were less encouraging. Oral glucose only elicited modest (gastrin and CCK) or no increase (secretin) of endogenous secretion of the known gut hormones. And the effect of isolated exogenous administration in physiologically relevant doses of for instance gastrin only stimulated insulin secretion to a minor extent [(44), see also Figure 1]. A third line obtained more success with two later identified gut hormones. In the early 1970's, first in the laboratory of Viktor Mutt in Stockholm, John Brown isolated GIP as an inhibitor of gastric acid secretion (46, 47). In subsequent studies, however, John Brown together with John Dupré showed that GIP is a potent releaser of insulin during hyperglycemia, but without effect in euglycemia (48). Thus, GIP was a glucose-dependent incretin and was accordingly renamed “glucose-dependent insulintropic polypeptide,” hence maintaining the acronym with the new name [for reviews, see also Creutzfeldt (49) and (50)]. The following quantitative studies of the incretin effect of GIP, however, suggested that GIP could not explain the entire gut hormonal effect on insulin secretion after oral glucose.

Then in the mid- and late 1980's, an additional gut hormone with incretin-activity surfaced. The background for the discovery was Unger and co-workers' RIA-observations in the 1960's that the intestinal mucosa expressed some glucagon-like immunoreactivity, which was different from the well-known pancreatic glucagon peptide; hence the name “gut glucagon” (51–53). Several laboratories in Europe and North America subsequently tried to identify the bioactivity and structure of gut glucagon peptides in the hope that one of them might be a missing incretin [for review, see for instance (54)]. An essential premise for success in this endeavor became the cloning and sequencing of mammalian glucagon genes by Graeme Bell and co-workers in 1983 (55, 56). The cDNA-deduced proglucagon structure revealed unequivocally that the prohormone in addition to the sequence of pancreatic glucagon contained the sequences of two novel *g*lucagon-*l*ike *p*eptides, which by Bell et al. were named GLP-1 and GLP-2. Both GLP's were expressed in the gut. GLP-1 as such had a modest insulin-releasing activity (57), but purification from gut extracts in the laboratories of Habener and Holst, respectively, showed that GLP-1 was also synthetized in a truncated (7-36) form with marked insulin-releasing effect (58–61). Moreover, the truncated GLP-1 turned out also to inhibit the secretion of pancreatic glucagon, which together with its insulinotropic effect (59, 60) counteracts the hyperglycemia in diabetes (62, 63). That GLP-1 moreover is a satiety signal that facilitates weight loss and— as shown later—ameliorates the cardiac function in diabetes has made GLP-1 an obvious drug target for treatment of type 2 diabetes mellitus. Several GLP-1-derived drugs are consequently now on the market and have been subject to comprehensive randomized and controlled trials [for recent reports, see (64–68)]. So far, so good for GLP-1 and GIP as incretins (2, 4). Essential milestones in first century of the history of the incretin concept are pinpointed in Table 1.

**Table 1:** Twelve milestones in the first century of the history of the incretin concept.

**Name(s)**	**Contribution**	**Year**	**References**
1. Mering and Minkowski	Pancreas as the site of diabetes	1889	(14)
2. Bayliss and Starling	Discovery of secretin; the first hormone	1902	(5)
3. Starling	A gut hormone may stimulate the endocrine pancreas	1905	(6)
4. La Barre and Still	Evidence of an insulinotropic gut hormone	1930	(22)
5. La Barre	Coining the word incretin	1932	(8)
6. Yalow and Berson	Invention of the radioimmunoassay	1960	(30)
7. McIntyre et al. and Elrick et al.	Demonstration of a glucose-dependent incretin mechanism	1964	(33, 34)
8. Unger et al.	Gut glucagon-like immunoreactivity	1966	(52)
9. Brown et al.	Identification of GIP	1971	(46, 47)
10. Dupré and Brown	GIP as an incretin	1973	(48)
11. Bell et al.	Identification of GLP-1	1983	(55, 56)
12. Habener et al. and Holst et al.	Truncated GLP-1 as an incretin	1987	(59, 60)

## A problem

While nobody questions the insulinotropic activities of GIP and GLP-1, it has become a problem that the present enthusiasm for the two hormones and not least for GLP-1-derived drugs has virtually suppressed supplementary ideas about additional incretin activity of other gut hormones. The problem reflects an old-fashioned and somewhat incorrect view on gastrointestinal endocrinology, not least among GLP-1 enthusiasts. There are different aspects to consider in this context.

**Figure 2:**
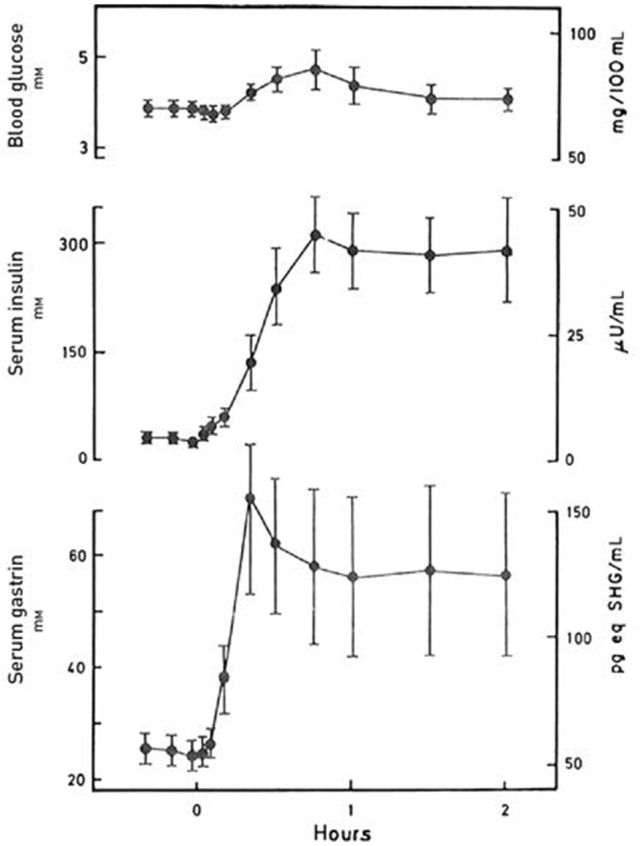
Blood glucose, serum insulin and serum gastrin concentrations during a protein-rich meal. The concentrations are indicated as mean ± SEM (*n* = 8). Data from Rehfeld & Stadil (44).

First, the original definition of incretin is as stated “any gut hormone, which under physiological circumstances stimulates the secretion of pancreatic hormones.” Indeed, several gastrointestinal hormones beyond GIP and GLP-1 stimulate insulin (see Table 2). For instance, gastrin accentuates glucose-stimulated insulin secretion significantly (Figure 1), and occasionally also glucagon secretion (37, 44). The effect of the other hormones administered exogenously alone in the fasting state may, however, be small and look trivial. But in combination with for instance EGF (epidermal growth factor), GLP-1 and/or during a meal (Figure 2), the effect may be significant as discussed in detail for instance for gastrin and cholecystokinin (37, 44, 69–73). Also the new gut hormone, xenin (74) displays promising incretin activities (75–77). And acute administration of PYY (1-36) as well as somatostatin inhibits insulin secretion. Moreover, examination of gut hormone receptors on the cell-membranes of pancreatic islet-cells is likely to show that a considerable number of gastrointestinal hormones directly influence the secretion of pancreatic hormones. For instance, Reubi et al. found a fairly abundant expression of gastrin and CCK receptors on human pancreatic islet cells (78).

**Table 2:** Examples of gastrointestinal neuroendocrine peptides that require (further) examination of their incretin activity*.

Adrenomedullin
Apelin
Calcitonin Gene-Related Peptide (CGRP)
Cholecystokinin
Galanin
Gastrin
Gastrin-Releasing Peptide (GRP)
Ghrelin
Leptin
Motilin
Neurotensin
Neuropeptide Y (NPY)
Obestatin
Opioids
Pituitary Adenylate Cyclase Polypeptide (PACAP)
Peptide YY (PYY)
Vasoactive Intestinal Polypeptide (VIP)
Xenin

*Full examination requires studies of the isolated effect on basal and stimulated islet-hormone secretion as well as studies of synergistic effects in combination with the other gastrointestinal hormones (including GIP and GLP-1).

Second, the old textbook-understanding of gastrointestinal endocrinology has been a “one-hormone-one-target” without functional overlap between the hormones: Gastrin regulated gastric acid secretion; secretin pancreatic bicarbonate secretion; CCK mainly gallbladder emptying; GIP only inhibition of gastric secretion; motilin intestinal motility etc. This understanding has in many ways turned out to be wrong and misleading. Today we know that the digestive tract is the largest and phylogenetically oldest endocrine organ in the body in which 30 different hormone genes are expressed and where the prohormones are cellularly processed to more than 100 bioactive peptides. Each hormone system has several targets both in and outside the gastrointestinal tract. And different hormones may simultaneously target the same organ and cells synergistically with both stimulatory and inhibitory signals. Moreover, the same enteroendocrine cell may express two or more different hormone genes. And the enteroendocrine cells for a given hormone are considerably more widespread in the gut than hitherto assumed. For instance gastrin/CCK_2_-receptor agonists are expressed all the way from the stomach to colorectal mucosa [for reviews, see for instance (79, 80)]. Thus, the limitation of incretin activity to only two peptides from the gastrointestinal tract may be somewhat naïve and old-fashioned.

Third, the delineation of incretin activity in such close relation to intake of glucose is also problematic. Of course, the concentrations of glucose in circulation are relevant in studies and discussions of insulin and glucagon secretion. But oral intake of 50 or 75 g pure glucose as used in the oral glucose tolerance tests is an unphysiological situation, which cannot be used to exclude gut hormones as incretins under normal physiological conditions. Several gut hormones respond as mentioned poorly to pure glucose. But many respond vividly to normal meals containing substantial amounts of protein, fat, and complex carbohydrates without major changes in blood glucose concentrations and under these circumstances stimulate islet-hormone secretion in synergy with other gut hormones (Figure 2). This in fact touches the fundamental role of the enteroendocrine system: That meals—depending on their composition—elicit variable polyphonies or rather symphonies of gastrointestinal hormones playing together to ensure optimal digestion and absorption of the food. This is the situation that is relevant for definition of general endocrine activities of the gut. Not only regarding incretin activity, but also other major cross-hormonal activities such as gastrointestinal motility, the inhibitory gastrone activities, satiety signaling etc., where several different hormones interact for the purpose of ensuring optimal nutrition.

## Conclusion

Incretin and the use of incretin hormones in diabetes therapy are old concepts with roots dating back to the second half of the nineteenth century. The history of incretin reflects a development characteristic for many lines of science with alternating progress and retrogressions. The situation for incretin today is based on decisive technical breakthroughs in disciplines such as peptide purification and sequencing; radioimmunoassay technology; cDNA cloning and sequencing; *in vitro* perfusion of endocrine organs; and *in vitro* synthesis of polypeptide constructs containing even three agonist epitopes. Probably, continued incretin research will reveal further integration of GIP and GLP-1 with additional gut peptides and provide a better and more comprehensive physiological understanding of the incretin concept. Such understanding may further the development of biomedical diagnosis and incretin therapy. This development is in fact already underway both in terms of GIP, GLP-1, and/or glucagon dual and triple receptor agonists (81–84), and—perhaps even more promising—dual or triple receptor agonists that combine GLP-1 analogs with analogs of some of the other gastrointestinal hormones (85–87).

## Author contributions

The author confirms being the sole contributor of this work and approved it for publication.

### Conflict of interest statement

The author declares that the research was conducted in the absence of any commercial or financial relationships that could be construed as a potential conflict of interest.
